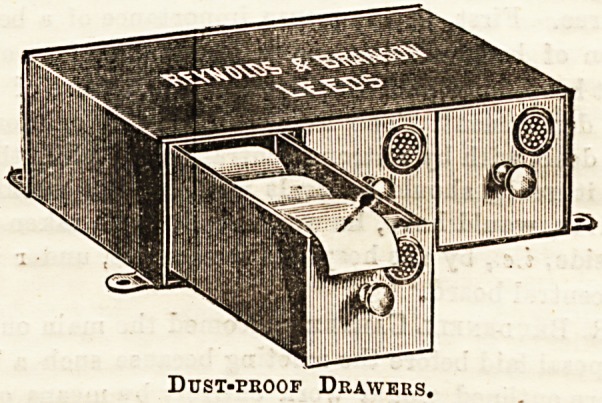# Practical Departments

**Published:** 1896-04-18

**Authors:** 


### PRACTICAL DEPARTMENTS.

### THE "FACILE PRINCEPS" BED.

A model of this bed, the invention of Mrs. Douglas, the wife of a doctor at Leamington, was exhibited at the British Medical Association meeting last summer, and then met with much appreciation from medical men and nurses. Suggestions made and criticisms given then have resulted in further improvements, and one of the perfected "Facile Princeps" beds is now to be seen at Mr. John Carter's, 6A, New Cavendish Street. The illustrations herewith give a fair idea of its character; the primary aim is to enable the changing of sheets to be carried through with as little disturbance as possible to the sick person. The bedstead itself is much the same as those in ordinary use, save that it must be of very strong make to bear the strain put upon it; for the sheet, which is a divided one, overlapping some inches in the centre, is strained tightly on rollers at the top and bottom of the frame, worked by wheels at one side. The rollers are supported by strong webbing bands, with straps and buckles, running over eight small wheels, four at each end of the bed. The sheet thus worked makes a kind of firm hammock, upon which the patient can be raised a good way from the mattress, fresh sheets laid beneath it, and then gently lowered, while, by means of two webbing bands and a simple mechanical contrivance, the soiled sheets are pulled apart and got rid of without any difficulty, the clean ones being afterwards secured to the rollers. Some objections have been urged to the divided sheet on the score of insecurity in the case of a heavy patient, although practical experience would seem to show that the arrangement is a perfectly safe one, as both sheets being perfectly taut there is practically no giving in the middle. But to meet this criticism Mrs. Douglas has thought out another plan by which a single sheet is used and pulled endwise from beneath the patient. It should be mentioned that the roller arrangement can be raised both ends at once or one at a time, so that an inclined position and also a sitting position (shown in the sketch, and worked by means of a fixed padded cushion) can be obtained if desired.

For use in private houses for operations, the patient, after being anæsthetised, can be raised to the height required, and a wooden board fixed firmly beneath, making a steady operating table at once. It is, perhaps, in the giving of baths in typhoid cases that this bed will be found of the greatest value, for macintosh sheets can be substituted for the linen ones; the patient raised while the bath is placed in position, and then lowered into it, the removal back again being quite easily accomplished. All the manipulations are effected with little or no exertion, one nurse being able, with the occasional assistance of some other person, to manage the changing of sheets and other movements. In this way it will doubtless be found to save much trouble and worry both to patient and nurse, and greatly minimise the lifting, which in heavy, helpless cases, is so serious a difficulty. The need for some such invention was brought home to Mrs. Douglas by practical acquaintance with illness, and the bed, as it now is, represents five years' thought and labour, for which many sick folk are likely in the future to be thankful.

### DUST-PROOF DRAWERS FOR DRESSINGS.

Messrs. Reynolds and Branson (Briggate, Leeds) have added to their list of ingenious devices "Dust-proof Self-closing Drawers" for keeping surgical dressings in. The illustration almost explains itself. The drawers are made in block tin, closing tightly with a spring at a slight touch, every part being made to fit closely. There is in each drawer

Dust-proof Drawers.

an air filter, loosely packed with a suitable filtering medium, which can be renewed whenever necessary. By this means the admission of dust into the receptacle is reduced to a minimum, and the dressings are successfully kept from all contact with impurities. The drawers can be obtained singly, or in sets of two or three or more, as in the drawing. They are neat and compact-looking, and are certain to find favour in hospital wards. The prices are, for one drawer, 7s. 6d.; set of four, 25s.; set of eight, £2 2s.

## Figures and Tables

**Figure f1:**
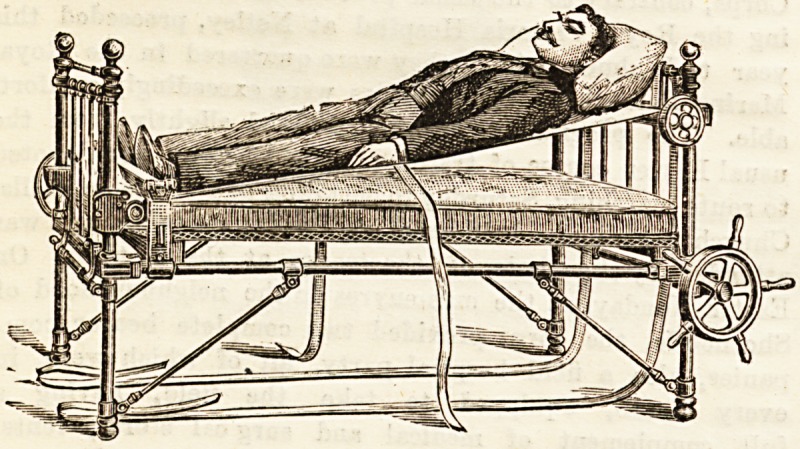


**Figure f2:**
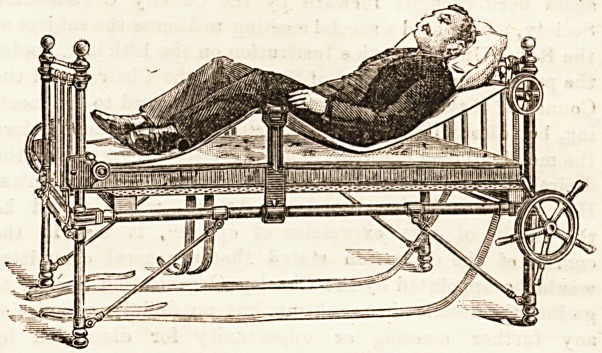


**Figure f3:**